# Von Willebrand Factor Abnormalities Studied in the Mouse Model: What We Learned about VWF Functions

**DOI:** 10.4084/MJHID.2013.047

**Published:** 2013-07-10

**Authors:** Caterina Casari, Peter J. Lenting, Olivier D. Christophe, Cécile V. Denis

**Affiliations:** 1 INSERM U770, Le Kremlin-Bicêtre, F-94276, France; 2 Univ Paris-Sud, UMR_S770, Le Kremlin-Bicêtre, F-94276, France

## Abstract

Up until recently, von Willebrand Factor (VWF) structure-function relationships have only been studied through *in vitro* approaches. A powerful technique known as hydrodynamic gene transfer, which allows transient expression of a transgene by mouse hepatocytes, has led to an important shift in VWF research. Indeed this approach has now enabled us to transiently express a number of VWF mutants in VWF-deficient mice in order to test the relative importance of specific residues in different aspects of VWF biology and functions in an *in vivo* setting. As a result, mice reproducing various types of von Willebrand disease have been generated, models that will be useful to test new therapies. This approach also allowed a more precise identification of the importance of VWF interaction with subendothelial collagens and with platelets receptors in hemostasis and thrombosis. The recent advances gathered from these studies as well as the pros and cons of the technique will be reviewed here.

Although von Willebrand disease (VWD) was first recognized as early as 1926 by Erik von Willebrand,^1^ it is only in the 1970 s that the protein responsible for this disease, *i.e.* von Willebrand factor was formally recognized as an independent entity not to be confused with coagulation factor VIII (FVIII). Other articles in this special issue have extensively developed all aspects related to VWF functions in hemostasis/thrombosis as well as beyond these fields and also the pathological aspects with regard to VWD. Some of these progresses were made possible thanks to the availability of animal models of VWD. Indeed, VWD has been described as naturally occurring in a number of different species such as pigs, dogs, cats, and mice.^2^,^3^ In addition, the progress made in the last 20 years in genetic manipulation has allowed the engineering of additional models of VWD especially murine models. A knock-out mouse deficient in VWF was generated and was shown to adequately model severe human VWD.^4^ This model has proven invaluable to study specific aspects of VWF functions and has uncovered many unsuspected leads, regenerating the research interest for this protein.^5^,^6^ However, after a few years, the limitations of the model became apparent. Indeed, it does not allow a more subtle analysis of the relative importance of the different VWF domains. When considering the large size of VWF and its capacity to interact with various ligands, the necessity to evaluate independently these interactions appeared increasingly crucial. In addition, the motivation to evaluate *in vivo* the effect of mutations detected in VWD patients, also fueled interest in gaining access to additional mouse models reproducing such defects. Using the homologous recombination technique to engineer genetically modified mice expressing mutated VWF (knock-in mice) would be the ideal approach but it is time-consuming, expensive and technically challenging. An alternative consists in using the hydrodynamic injection technique, which allows transient expression of a transgene by mouse hepatocytes. This approach has now been implemented with success in several laboratories and in this article, we will review the new information that was gathered from its use.

## Hydrodynamic injection: application to VWF expression

**Figure 1:**
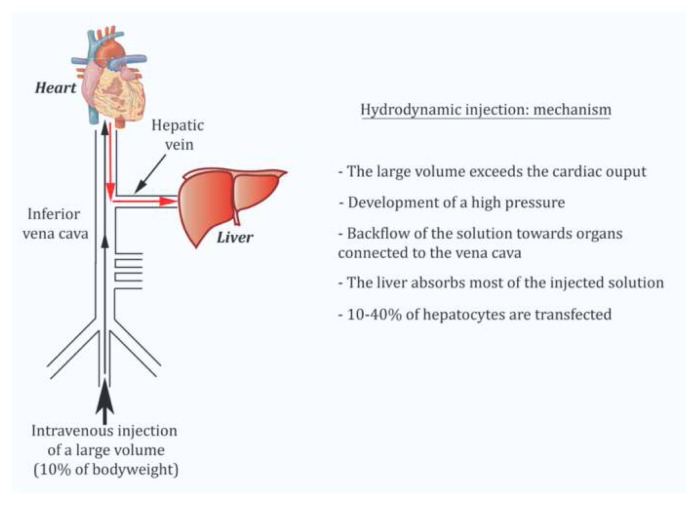
Description of the mechanism leading to hepatocyte-derived expression of foreign proteins following hydrodynamic injection with plasmid DNA.

Hydrodynamic injection is akin to an *in vivo* transfection method (Figure 1). It consists in a rapid injection of a large volume of plasmid DNA without any other viral or non-viral vector in the lateral tail vein of mice.^7^ The volume and speed of injection have been shown to strongly influence the efficiency of gene delivery to hepatocytes. In order to be fully optimal, injection should be completed within 5 seconds with a volume of injection corresponding to 10% of the body weight of the mouse (*i.e.* 2 ml for a 20 g mouse).^8^ After undergoing a successful injection, the mice go through an apathetic phase (shock) that is transient. In C57BL/6 mice, recovery occurs after approximately 20–30 minutes and the survival rate is 100%. The following mechanism has been proposed to explain hepatocytes transfection: by exceeding cardiac capacity, the large DNA volume accumulates in the lower vena cava. This leads to a sharp increase in venous blood pressure and a backflow of the solution towards organs connected to the vena cava. The liver has the most extensive structure and absorbs the majority of the volume injected (>90%). The elevated pressure generated by hydrodynamic injection opens sinusoid fenestrae allowing the solution to reach hepatocytes. On average, 10–40% of hepatocytes are transfected. It is rather amazing that despite the traumatic aspect of the technique, liver biological parameters are only slightly modified and on a temporary basis. Indeed, only levels of serum alanine aminotransferase and of aspartate aminotransferase are elevated after hydrodynamic gene delivery (4–20 fold according to different studies) but they return to baseline three days later. Histological studies also showed that less than 5% of the liver has suffered from hepatic damage. Other parameters such as alkaline phosphatase, total bilirubin, albumin, total proteins and Na^+^, K^+^ and Cl^−^ ions are not modified.^7^

**Figure 2:**
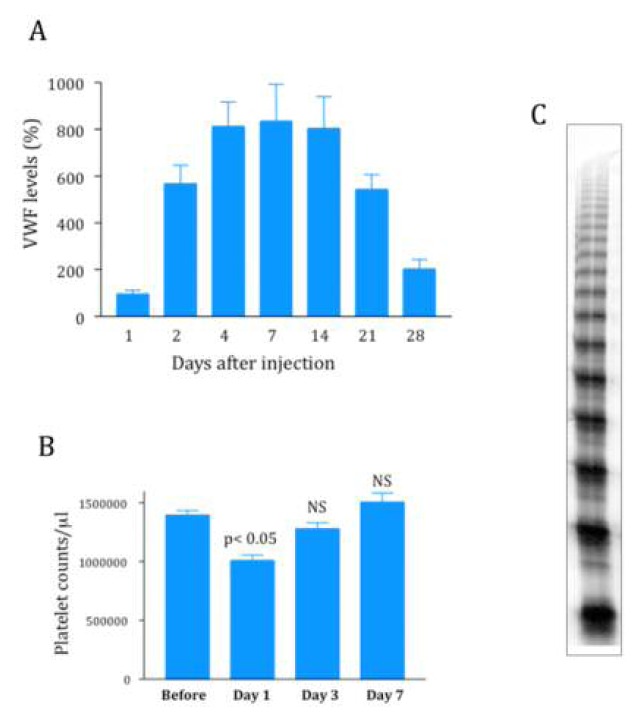
Characteristics of VWF expression after hydrodynamic gene-transfer in VWF-deficient mice. A: Injection of 50 μg of plasmid pLIVE-mVWF cDNA resulted in plasmatic expression levels around 800% (100% being the plasma VWF levels measured in wild-type mice) and was stable for about two weeks. Expression levels can be highly operator-dependent. B: Platelet counts were recorded for a few days following hydrodynamic gene transfer and were shown to slightly decrease 24 hours after injection but were back to pre-injection levels after 3 days. C: Multimeric profile of plasma VWF produced by hepatocytes following hydrodynamic gene transfer.

This technique has now been used successfully to express functionally active VWF in VWF-deficient mice.^9^ Several potential hurdles had to be overcome before such a result could be obtained. First, there was a species-barrier since human VWF interacts poorly with its platelet receptor glycoprotein (GP) Ibα, in mice. As a consequence, only murine VWF (mVWF) cDNA can be used. mVWF cDNA was therefore subcloned in different vectors, either with a CMV promoter^10^ or with a liver-specific promoter (murine albumin or α1-antitrypsin).^11^,^12^ In all cases, high pressure injection of 50–100 μg of mVWF cDNA resulted in high levels of expression of VWF antigen in plasma of VWF-deficient mice (Figure 2). Vectors with liver-specific promoters allowed relatively long-term expression (a few weeks compared to a couple of days for vectors with a CMV promoter). A temporary decrease in platelet counts could also be observed but was not apparent anymore after 72 hours (Figure 2). Immunostaining showed that, as expected, VWF is expressed by hepatocytes and not by endothelial cells.^13^ This hepatocyte-expression of VWF represented the second hurdle since it was not clear whether this cell type had the necessary machinery to multimerize VWF, a critical criteria for VWF biological activity. Fortunately, we were able to show that this hepatocyte-derived VWF was indeed multimerized (Figure 2) and that it also had the capacity to bind endogenous FVIII, leading to restoration of plasma FVIII levels, which are normally strongly reduced in VWF-deficient mice. Finally the ultimate proof of the functional quality of liver-expressed mVWF was demonstrated by its capacity to correct bleeding time in VWF-deficient mice.^10^ In addition, thrombus formation after ferric-chloride induced injury was also restored in the knockout mice expressing plasma mVWF.^11^,^12^

These results show that plasma VWF is sufficient to support efficient hemostasis and thrombosis in the mouse, indicating that liver-targeted gene therapy can be considered as a viable treatment alternative for severe VWD. This model also allows the direct comparison of hemostatic capacities of wild-type versus mutant VWF.

## Analysis of VWF variants carrying mutations in binding domains to different ligands

In order to play its role in hemostasis, VWF binds to a number of different ligands, among which two platelet receptors, platelet GPIbα and GPIIbIIIa as well as subendothelial collagens.^14^ Thanks to many *in vitro* biochemical studies, the residues on human VWF involved in its binding to these ligands have been precisely identified.^15^–^17^ Considering that these residues were perfectly conserved in mVWF, we hypothesized that the same sequences were also involved in mVWF binding to its murine ligands on platelets and in the vessel wall. We therefore mutated these residues in order to abolish binding to the different receptors.^10^ To abolish mVWF binding to murine GPIbα, we mutated the lysine residue in position 1362 to an alanine. Binding of mVWF to mGPIIbIIIa was suppressed by mutating the aspartic acid residue in position 2509 to glycine (RGD sequence converted to RGG sequence). Finally to abolish binding of mVWF to collagens, three residues were mutated simultaneously: aspartic acid 1742, serine 1783 and histidine 1786 were all converted to alanine. The lack of interaction of these different VWF variants to their respective ligands was confirmed *in vitro*.^10^ Hydrodynamic gene transfer of these three mutants showed that 1) comparable plasma mVWF expression levels were obtained, suggesting that the mutations do not alter VWF biosynthesis and 2) plasma VWF was correctly multimerized for all mutants, suggesting that the mutations do not interfere with the multimerization process.

### Effect of mutations on bleeding time

Patients with VWD type 2A or 2M already made it clear that a form of VWF that could not interact with platelet GPIbα lead to bleeding symptoms.^18^ Injection of the mVWF cDNA carrying the K1362A mutation in VWF-deficient mice confirmed this observation.^10^ In contrast to mice injected with wild-type mVWF cDNA, these mice could not control their bleeding time in the tail clip assay and kept bleeding indefinitely, similar to untreated mice. In contrast to the expected bleeding phenotype predicted for the VWF mutant unable to bind GPIbα, no real indication of what to anticipate for lack of binding to collagens or to GPIIbIIIa could be deducted from VWD patients. Indeed, no patient with a mutation in the GPIIbIIIa binding domain has been reported so far and only very few patients with mutations in the collagen-binding region were described at the beginning of this study. Interestingly, VWF-deficient mice injected with either the collagen binding mutant or the RGG mVWF cDNAs were able to control their bleeding as well as wild-type mVWF-treated mice, indicating that these interactions are not absolutely required for the physiological process of hemostasis in the mouse model.^10^

### Effect of mutations on thrombosis

Since a defect or lack thereof, in functional activity in the bleeding model is not necessarily representative of the complex role played by VWF in hemostasis/thrombosis, we also extended our examination of these mutations in an *in vivo* thrombosis model initiated by ferric-chloride exposure of mesenteric vessels.^12^ We first showed that injection of wild-type mVWF cDNA in VWF-deficient mice was able to restore thrombus formation and vessel occlusion with a kinetic very similar to the one obtained with wild-type mice. Mice expressing the GPIbα-binding mutant did not form occlusive thrombi, similar to untreated mice. This result, added to the incapacity of this mutant to correct bleeding time, confirms the critical importance of the VWF-GPIbα interaction in maintaining efficient hemostasis/thrombosis. More surprisingly, mice expressing the collagen binding mutant showed altered thrombosis with significantly delayed vessel occlusion (30 min versus 16 min in wild-type VWF-expressing mice).^12^ However, despite this delay, the mice were able to form stable thrombi well anchored to the vessel wall. In contrast, mice expressing the GPIIb/IIIa binding mutant were more severely affected. Less than 40% of the mice expressing this mutant were able to reach occlusive thrombosis. Mice formed thrombi that were less compact and which were not efficient to stop blood flow. Portions of these thrombi constantly embolized, preventing further growth of the thrombus. This result emphasized the critical importance of the VWF-GPIIbIIIa interaction in platelet-platelet firm adhesion at high shear rates.

Altogether, results from the bleeding time as well as the thrombosis model experiments suggested that VWF interactions with fibrillar collagens and with GPIIbIIIa are more important in the pathological thrombotic process than in the physiological process of hemostasis.

### Effect of mutations on stroke

The same three VWF mutants were also tested in an ischemic stroke model in mice in order to establish which VWF interactions were important in this model.^19^ Stroke was induced by transient middle cerebral artery occlusion. Previous reports had already described a protection against stroke in mice deficient in VWF, while replenishing the VWF plasma compartment by hydrodynamic gene transfer with wild-type mVWF cDNA totally restored the susceptibility of the mice to cerebral ischemia.^11^ Analysis of VWF-deficient mice hydrodynamically injected with the mVWF cDNAs mutated in the binding sites to fibrillar collagens, GPIbα or GPIIbIIIa revealed that interaction with GPIbα or collagens were involved in stroke development while interaction with GPIIbIIIa was not playing any role. Indeed the brain infarct volumes were significantly smaller in mice expressing the collagen mutant or the GPIbα mutant compared to mice expressing wild-type or GPIIbIIIa mutant VWF. Along the same line, the functional outcome as measured by neurological Bederson score or the grip test score were significantly higher in untreated VWF−/− mice or VWF−/− mice expressing the collagen or GPIbα mutants compared to wild-type or GPIIbIIIa-treated mice.^19^ Interestingly this result not only contributes to our understanding of the molecular mechanisms involved in ischemic brain injury but by showing that VWF-GPIIbIIIa interaction is not involved in this process it also sheds some light on our comprehension of the lack of effectiveness of GPIIbIIIa blockade in experimental and clinical stroke.^20^

### Application for anti-thombotic treatment

The observation that VWF interactions with fibrillar collagen or with platelet GPIIbIIIa may be more important for thrombosis than for hemostasis makes these axes interesting for new antithrombotic therapies.^12^ Monoclonal antibodies directed against human VWF and specifically targeting these interactions have been described in the litterature.^21^–^23^ However, so far the absence of accessible animal models has made it difficult to test the *in vivo* efficacy of such tools targeting human VWF. To bypass this species barrier, we have developed a human-murine VWF chimera where the human VWF A1 domain, *i.e.* the domain containing the GPIbα binding region, is replaced by its murine counterpart.^24^ This chimeric molecule proved to be functional after *in vivo* expression (through hydrodynamic injection) in VWF-deficient mice since it supported occlusive thrombosis after ferric-chloride-induced vascular injury. Being predominantly human, the chimera still contains the epitopes recognized by monoclonal antibodies targeting the VWF-collagen of VWF-GPIIbIIIa interactions. Hydrodynamic gene transfer was therefore performed to express this chimeric molecule in VWF-deficient mice and the inhibition of thrombus formation by monoclonal antibodies was measured in the mice. Inhibition of the VWF-collagen or VWF-GPIIbIIIa interactions proved efficient to reduce thrombosis in arterioles. In particular, one of the antibodies inhibiting VWF-collagen interaction was the most powerful, preventing vessel occlusion in 7 mice out of 8 tested.^24^ Hydrodynamic gene transfer of this human-murine VWF chimera thus proved to be an efficient manner to test various agents targeting human VWF in an *in vivo* system.

## Analysis of mice reproducing different subtypes of human VWD

VWD is characterized by quantitative and/or qualitative abnormalities in VWF with heterogeneous clinical manifestations.^18^,^25^ Our understanding of this disease has recently improved based on some large multicenter studies in Europe, Canada and the US^26^,^27^ but also on the use of murine models of VWD. Indeed, the hydrodynamic gene transfer has now allowed detailed *in vivo* genotype-phenotype analysis for a number of VWD types.

### Type 1 VWD

Although type 1 VWD, encompassing mild to moderate reduction in VWF levels, is by far the most common VWD type, its molecular pathogenesis is also the least well understood. Large multicenter studies in Europe, Canada and United States have recently contributed to shed light on the complex mechanisms at the basis of type 1 VWD.^26^–^28^ These may involve defective RNA or protein synthesis, intracellular protein degradation, impaired secretion, rapid plasma clearance, all of which can result from either a mutation in the VWF gene or the presence of modifier genes interfering with VWF biology.^3^,^29^,^30^ What has become increasingly clear from these studies, is the absolute need for multiple approaches to identify these complex and sometimes multiple mechanisms. Cellular studies are extremely useful to identify aspects related to synthesis and secretion, especially now that the possibility to grow blood outgrowth endothelial cells (BOECs) directly from the patient s blood is available.^31^–^34^ However, when it comes to plasma clearance or detection of a gene modifier, a more complex organism is needed. No transgenic type 1 VWD murine model has been generated so far. However, two common type 1 VWD mutations have been expressed in VWF-deficient mice by hydrodynamic gene transfer: the p.R1205H Vicenza mutation and the p.Y1584C mutation.^35^ Results confirmed the aptitude of this *in vivo* murine expression system to fully recapitulate a complex clinical picture already observed in VWD patients. Indeed, both in the homozygous and heterozygous states, mice expressing either of these two mutants displayed lower VWF antigen levels compared to wild-type VWF expressing mice. Mice expressing the p.R1205H Vicenza mutant had an increased VWF propeptide/VWF antigen ratio, a feature indicating accelerated clearance of the protein. This confirms results found in patients as well as results obtained in a murine model with injection of the recombinant protein.^36^,^37^ Mice expressing the p.Y1584C mutant displayed increased ADAMTS13 proteolysis, also supporting studies performed with patients plasma.^38^,^39^ This model also allowed the authors to study the procoagulant function of the mutant proteins in *in vivo* thrombosis experiments and to do so at a fixed VWF antigen plasma concentration. This approach has the advantage to evaluate the inherent capacity of the mutant protein to promote thrombus formation, independently of the quantitative anomaly. Results showed that the p.R1205H VWF exerts a normal hemostatic function, suggesting that the associated defect in patients is purely of a quantitative nature while the p.Y1584C VWF displays a lower capacity to support thrombus formation, resembling a mild type 2A VWD phenotype, probably due to the increased ADAMTS13 cleavage.^35^

### Type 2M VWD: collagen-binding mutants

So far, all the VWF mutations that have been tested using the hydrodynamic injection procedure were introduced in the murine VWF sequence. The mouse VWF amino acid sequence has 91% homology to that of human VWF,^40^ allowing replacement of the same residue in the murine system as the one affected in the patients. As mentioned earlier, only murine VWF is hemostatically active in the mouse, preventing use of human VWF. However, since we had generated an active human-murine VWF chimera (hVWFmA1) in order to test the anti-thrombotic effect of monoclonal antibodies inhibiting specific VWF functions,^24^ we also used the same construct to introduce VWF mutations present in the A3 domain and detected in patients with VWD.^41^ Mutations in the VWF A3 domain have not been described as frequently as mutations in the platelet-binding domain (the A1 domain) but they are now being increasingly recognized.^42^–^44^ These mutations usually lead to deficient VWF-collagen interaction and mild/moderate bleeding symptoms. Two such mutations detected through the French National Reference Center for VWD were introduced in the hVWFmA1 cDNA and injected to VWF-deficient mice using hydrodynamic gene transfer: p.L1696R and p.P1824H. In contrast to above-mentioned studies using type 2B and type 1 mutants where the *in vivo* studies were performed in a second step following extensive *in vitro* analysis, the mutants were here studied in parallel *in vitro* and *in vivo* in order to get a complete picture of the functional defects associated with the mutations. This dual approach proved extremely useful to identify the dominant-negative effect of the p.L1696R mutation on VWF expression levels in patients and in mice expressing the mutation in a heterozygous manner, compared to cellular expression systems. This result raised the possibility that the mutant protein was cleared more rapidly than wild-type VWF *in vivo*, a feature that cannot be detected *in vitro*. Mice expressing the second A3 domain mutant, p.P1824H-VWF also displayed low expression VWF levels, but the probable underlying cause, intracellular retention was this time detected both in the *in vitro* and the *in vivo* expression systems.^41^ For both mutants, the thrombotic response in arterioles of mice was strongly decreased, but the low expression levels did not allow concluding as whether this effect should be attributed to the quantitative defect or to the qualitative defect.

### Type 2B VWD

Type 2B VWD is characterized by gain-of-function mutations in VWF A1 domain, resulting not only in an increased affinity for GPIbα but also in an increased susceptibility to ADAMTS13.^25^,^45^ VWF carrying common type 2B mutations, p.V1316M, p.R1306Q, p.R1306W or p.R1341Q.^25^,^45^,^46^ were expressed in VWF-deficient mice by hydrodynamic gene transfer to generate type 2B VWD murine models. A clear mutation-dependent modulation of disease severity was described, with variable thrombocytopenia and loss of high molecular weight VWF multimers, presence of circulating platelet aggregates and of giant platelets and defective hemostasis and thrombosis.^47^,^48^ Strikingly, these features parallel remarkably well with the phenotype of the patients carrying similar mutations.^46^ It therefore appears that these generated transient mouse models are sensitive enough to recapitulate the myriad of defects associated with human type 2B VWD and to detect subtle differences in phenotype. Such models have been instrumental in establishing that the loss of high molecular weight multimers *in vivo* is caused by the enhanced susceptibility to ADAMTS13 proteolysis rather than preferential adsorption of the high multimers onto platelets.^48^ These type 2B VWD mouse models will be very useful in the future, not only to understand additional factors involved in this VWD type such as the mechanisms underlying thrombocytopenia but they also represent very useful tools to test new potential treatments.

## Study of ADAMTS13-cleavage mutants

Cleavage of VWF by ADAMTS13 is an important step in the regulation of VWF activity and any perturbation in the delicate balance between too little or too much cleavage leads to pathologic consequences: hemorrhagic when cleavage is excessive as is the case for VWD type 2A or thrombotic consequences when cleavage is insufficient or lacking as is the case in thrombotic thrombocytopenic purpura (TTP). In order to evaluate the *in vivo* consequences of interfering with VWF sequences involved in ADAMTS13 cleavage, Pruss and colleagues used hydrodynamic gene transfer to generate transient murine models expressing VWF carrying the p.R1597W mutation, the most common type 2A VWD mutation or VWF carrying mutations at the ADAMTS13 cleavage site p.Y1605A/M1606A.^49^ Analysis of the VWF multimeric profile of these mice confirmed a loss of high molecular weight multimers in p.R1597-VWF expressing mice and in contrast, a profile with higher molecular weight bands in p.Y1605A/M1606A-VWF expressing mice. This effect was even further amplified when full-length ADAMTS13 cDNA was co-injected with VWF cDNA in order to compensate for the presence of the truncated ADAMTS13 form that is naturally expressed in the C57Bl/6 mouse strain^50^ and that is not fully active *in vivo*.^51^,^52^ Interestingly, mice expressing the cleavage-resistant mutant, even at high concentrations, did not display any sign of TTP, similar to mice deficient in ADAMTS13.^53^,^54^ This result confirms that additional triggering events are necessary to induce TTP, at least in the murine model. The most striking observations were obtained in the *in vivo* thrombosis model induced by ferric-chloride injury of the cremaster muscle microcirculation. Indeed, initial platelet adhesion was not decreased in mice expressing p.R1597-VWF compared to mice expressing wild-type VWF, despite the absence of high molecular weight multimers in the former, suggesting that these most active forms are not absolutely required for this initial platelet adhesion step.^49^ However, time to occlusion was severely prolonged in the p.R1597-VWF expressing mice. Such results are reminiscent of results observed with VWF-deficient mice where platelet adhesion and thrombus formation are still observed but vessel occlusion is a rare event due to the absolute requirement for VWF^55^ and most likely fully multimerized VWF in order to close the last portion of the vessel where blood circulates under conditions of very high shear stress. Perhaps even more surprising was the observation that initial platelet adhesion was increased in mice expressing the p.Y1605A/M1606A-VWF mutant while vessel occlusion was lacking, a very puzzling result that remains largely unexplained so far.^49^

## Study of VWF O-glycome through hydrodynamic gene transfer

VWF is a heavily glycosylated proteins with 12 N-linked and 10 O-linked glycosylation sites per mature monomer,^56^ accounting for approximately 20% of the molecular weight of the protein. The role of N-glycans in VWF biology and function has been extensively studied^57^–^60^ in contrast to the role of O-glycans. Assessing the role of such glycans using hydrodynamic gene transfer is clearly a challenge since VWF is produced in hepatocytes and not by its physiologic production sites, endothelial cells and megakaryocytes. However, heterologous cell lines such as HEK293 have been commonly used to study VWF glycosylation and brought valuable information despite the fact that they can be similarly questioned in terms of relevance. In our team, we chose the option to investigate the role of VWF O-glycans by mutating individual sites or clusters of such glycans in the context of the murine system. First, we compared hepatocyte-derived VWF to normal plasma VWF for its ability to bind to a number of lectins and found that a similar extent of terminal galactose and sialic acids were exposed on endothelial and hepatic-derived VWF.^61^ Similarly, we showed that VWF produced by hepatocytes is not fundamentally different from endothelial-derived VWF in terms of peanut agglutinin recognition, a lectin recognizing T-antigen, the major O-linked glycan present in VWF.^62^,^63^ We therefore felt confident that we could use hydrodynamic gene transfer to study the functional role of VWF O-glycans. Mice carrying mutations in one or several potential VWF O-glycosylation sites were thus generated and studied in terms of VWF expression levels, multimerization, functionality and clearance. Our results showed that O-glycosylations are not absolutely required for VWF biosynthesis and multimerization. However, two residues T1255 and T1256 appeared to be of particular importance since they are involved in the maintenance of VWF plasma levels as well as in the capacity of VWF to support hemostasis. A third residue, S1486, was also shown to play an important role in VWF function.^61^

## Conclusion

In the last few years, a number of articles using hydrodynamic gene transfer to study VWF variants have been published by different teams, allowing us to acquire a good perception on the advantages and of course on the drawbacks of this very powerful approach. The benefits are rather obvious, allowing evaluation of VWF mutations in a complete organism more simply than by using *in vitro* transfections, making it a potential first line approach to screen such mutations. This has led to very interesting results, uncovering the relative importance of some mutations in the thrombotic process versus the hemostastic process. Furthermore, the model proved sensitive enough to detect subtle differences in phenotype, improving our comprehension of some patient s bleeding symptoms. It is rather paradoxical that such developments were obtained while VWF is at first glance clearly not an ideal candidate target for hydrodynamic gene transfer. Indeed a number of important challenges could have hampered research progress and they did so for a while. First, VWF needs to be multimerized to be active, an additional burden for the hepatocytes. Second, human VWF is not functional in the mouse, leading to the necessity to introduce mutations in the murine cDNA, or in a chimeric human-murine VWF construct. Third, with hydrodynamic injection only the VWF plasma compartment is replenished, leaving endothelial cells, the subendothelium and platelets, VWF-free. This aspect is particularly relevant when testing the ability of mutant-VWF to correct bleeding time in mice. In our hands, we have noticed that an expression level of around 300% of VWF needs to be reached in order to obtain bleeding time correction, probably as a result of the absence of VWF in compartments besides plasma and also possibly to a slight decrease in high molecular weight multimer bands. Despite all these potential issues, hydrodynamic gene transfer appears like an extremely valuable approach to investigate a complex phenotype associated with VWF modifications, ranging from biosynthesis to function to clearance of the variant molecule.

## Figures and Tables

**Table 1:** List of VWF mutations tested in vivo using hydrodynamic gene transfer in VWF-deficient mice

Mutation	Type of mutation	Impaired Biosynthesis	Impaired multimerization	Accelerated clearance	Capacity to correct bleeding time	Capacity to promote platelet adhesion in arterioles	Capacity to promote arterial occlusion	Capacity to protect from stroke	*Ref*
**K1362A**	GPIb binding site	No	No	No	No	No	No	Yes	*10,12,19*
**D1742A/S1783A/H1786A**	Collagen binding site	No	No	No	Yes	Yes	Decreased	Yes	*10,12,19*
**D2509G**	GPIIbIIIa binding site	No	No	No	Yes	Yes	Decreased	No	*10,12,19*
**R1205H**	Type 1 VWD	Possibly	No	Yes	ND	Yes	Yes	ND	*35*
**P1584C**	Type 1 VWD	Possibly	Yes	No	ND	Decreased	Decreased	ND	*35*
**L1696R**	Type 2M VWD	Yes	ND	ND	ND	Yes	Decreased	ND	*41*
**P1824H**	Type 2M VWD	Yes	ND	ND	ND	Yes	Decreased	ND	*41*
**V1316M**	Type 2B VWD	No	Yes	Yes	No	No	No	ND	*47,48*
**R1306Q**	Type 2B VWD	No	Yes	Yes	No	Decreased	No	ND	*48*
**R1306W**	Type 2B VWD	No	Yes	ND	ND	Decreased	No	ND	*47*
**R1341Q**	Type 2B VWD	No	Yes	ND	ND	Decreased	No	ND	*46*
**R1597W**	Type 2A VWD	No	Yes	Yes	ND	Yes	Decreased	ND	*49*
**Y1605A-M1606A**	ADAMTS13-cleavage site	No	Yes (increased)	Yes	ND	Increased	Decreased	ND	*49*
**T1248A**	O-gly site	No	No	No	Yes	ND	ND	ND	*61*
**T1255A**	O-gly site	No	No	No	Decreased	ND	ND	ND	*61*
**T1256A**	O-gly site	No	No	No	Decreased	ND	ND	ND	*61*
**T1255A/T1256A**	O-gly site	Yes	No	Yes	No	ND	ND	ND	*61*
**T1468A**	O-gly site	No	No	No	Yes	ND	ND	ND	*61*
**T1477A**	O-gly site	No	No	No	Yes	ND	ND	ND	*61*
**S1486A**	O-gly site	No	No	No	Decreased	ND	ND	ND	*61*
**T1487A**	O-gly site	No	No	No	Yes	ND	ND	ND	*61*
**T1679A**	O-gly site	No	No	No	Yes	ND	ND		*61*
**T2298A**	O-gly site	No	No	No	Yes	ND	ND	ND	*61*

All mutations were introduced in murine VWF cDNA and expressed in VWF-deficient mice. The phenotype of the mice expressing the mutants was compared to the phenotype of mice hydrodynamically injected with wild-type murine VWF cDNA. ND indicates not done.
